# Characterizing the wheat (*Triticum aestivum* L.) phosphate transporter gene family and analyzing expression patterns in response to low phosphorus stress during the seedling stage

**DOI:** 10.3389/fpls.2025.1531642

**Published:** 2025-03-27

**Authors:** Meini Song, Pengcheng Li, Lirong Yao, Chengdao Li, Erjing Si, Baochun Li, Yaxiong Meng, Xiaole Ma, Ke Yang, Hong Zhang, Xunwu Shang, Huajun Wang, Juncheng Wang

**Affiliations:** ^1^ State Key Lab of Aridland Crop Science / Gansu Key Lab of Crop Improvement and Germplasm Enhancement, Lanzhou, China; ^2^ Department of Crop Genetics and Breeding, College of Agronomy, Gansu Agricultural University, Lanzhou, China; ^3^ Western Barley Genetics Alliance, College of Science, Health, Engineering and Education, Murdoch University, Murdoch, WA, Australia; ^4^ Department of Botany, College of Life Sciences and Technology, Gansu Agricultural University, Lanzhou, China

**Keywords:** *wheat (Triticum aestivum* L.), phosphate transporter protein, *PHT* gene family, gene expression, low phosphorus stress

## Abstract

**Introduction:**

Inorganic phosphorus (Pi) is an indispensable nutrient for plant growth, with phosphate transporter proteins (PHTs) having key roles in Pi uptake, transport, and signal transduction in plants. However, a systematic and comprehensive genomic analysis of the wheat *PHT* family (covering *PHT1-5* and *PHO1*) is lacking.

**Methods:**

In view of this, we successfully identified 180 *Triticum aestivum PHT* (*TaPHT*) members in 6 PHT families using bioinformatics, and performed in-depth phylogenetic analyses between these protein sequences and *PHT* family proteins from *Arabidopsis thaliana* and an important rice crop.

**Results:**

We observed that the *TaPHT* family could be subdivided into 6 phylogenetic clusters, specifically including 46 *TaPHT1*, 3 *TaPHT2*, 65 *TaPHT3*, 22 *TaPHT4*, 14 *TaPHT5*, and 30 *TaPHO1* members. We also comprehensively profiled the phylogenetic relationships, structural features, conserved motifs, chromosomal localization, cis-acting elements and subcellular localization of these members. These features showed a high degree of conservation within each subfamily. In particular, in the 2000 bp sequence upstream of the *TaPHT* genes, we identified multiple cis-acting elements closely related to Pi responses, such as P1BS (PHR1 binding site), MBS (MYB binding site), and a W-box (WRKY binding site), which suggested that *TaPHT* genes were possibly involved in Pi signaling pathways. We screened 24 *TaPHT* genes by qRT-PCR (real-time quantitative PCR) and investigated their expression in roots and shoots of two wheat cultivars (Pi efficient material SW2 and Pi inefficient material SW14) under low Pi stress conditions. All genes showed up-regulated expression patterns associated with Pi nutritional status, with relative gene expression generally higher in the SW2 cultivar when compared to SW14. Particularly noteworthy was that *TaPHT1;36* in the SW2 cultivar showed high and relative stable expression in wheat roots. Combining our bioinformatics and relative gene expression analyses, we preliminarily screened *TaPHT1;36* as a candidate gene for low Pi tolerance and further confirmed its subcellular localization.

**Discussion:**

Our work not only identified important *TaPHT* family roles in coping with low Pi stress, but it also provides a functional research basis and candidate gene resource for solving Pi deficiency-related problems.

## 1 Introduction

Food security represents a significant global challenge, one that is fundamental to human survival and development (Cordell et al., 2009). As the global population continues to grow, the inelastic demand for food is so too increasing. Inorganic phosphorus (Pi) is a major nutrient required for crop growth and development. It is vital for living organisms, serving as a nucleic acid and cell membrane components and participating in several key biological processes (Muchhal et al., 1996; Westheimer, 1987). However, the majority of phosphates are rapidly immobilized following phosphate fertilizer application to soils, making them unavailable for absorption and use by crops (Davies et al., 2002). Consequently, specialized transporter proteins are required to facilitate Pi transport from the soil to plant roots for uptake (Wang et al., 2019b).

Phosphate transporter proteins (PHTs) are responsible for Pi uptake and transport in plants. They are typically divided into 6 different subclasses based on sequence similarity, structure, and function, and are referred to as *PHT1*-*PHT5* and *PHO1* (Ahmad et al., 2021; Rui et al., 2023). Since the earliest identification of the high-affinity *PHT* gene, *PHO84*, in yeast, there has been a significant increase in *PHT* gene numbers identified in *Arabidopsis thaliana* (Muchhal et al., 1996), *Oryza sativa* (Paszkowski et al., 2002), *Malus domestica* (Sun et al., 2017), *Populus simonii* (Zhang et al., 2016), *Medicago truncatula* (Harrison et al., 2002), *Solanum tuberosum* (Liu et al., 2017), *Lupinus albus* (Aslam et al., 2022), *Spirodela polyrhiza* (Zhao et al., 2021b), *Brassica napus* (Yang et al., 2020), *Capsicum annuum* (Ahmad et al., 2021) and *Saccharum* (Yang et al., 2020), amongst others.

*PHT1* is one of the most studied subclasses in the *PHT* family, and is notably characterized by 12 transmembrane structural domains (Ullrich-Eberius et al., 1981). *PHT2* is localized in the inner chloroplast membrane and is mainly expressed in green tissues and roots, with its main function being phosphate transport within the leaf (Daram et al., 1999; Harrison et al., 2002). As the first identified mitochondrial Pi transporter gene, *PHT3* is highly conserved among the family and is essential for maintaining Pi homeostasis in plants under adverse conditions (Rausch and Bucher, 2002; Takabatake et al., 1999). *PHT4* is primarily localized to the plasma membrane, periplasm, and Golgi apparatus, and plays a key role in Pi transport (Guo et al., 2008; Wan et al., 2020). *PHT5*, in addition to its role in these organelles, is involved in several biological processes. It is located in vesicle membranes and has important roles regulating Pi homeostasis in plants, which regulates Pi storage and acclimatization in vesicles (Liu et al., 2016). *PHO1* is expressed in specific patterns in plants, predominantly in stellate cells in roots and the lower portion of hypocotyls. At these locations, *PHO1* facilitates effective Pi transport from root epidermal cells to the xylem and back to the roots via the phloem, thereby ensuring effective Pi recycling and use in plants (Poirier and Bucher, 2002; Ticconi et al., 2001).

Wheat (*Triticum aestivum* L.) is a major global food crop and essential for food production and security (Godfray et al., 2010). To enhance wheat yields, substantial Pi fertilizers are used to facilitate maturation and augment agronomic quality (Shukla et al., 2016). Pi is readily immobilized and has low mobility in soil, which impedes efficient Pi absorption in wheat. This causes Pi deficiency which reduces wheat yields and quality. Phosphate transporters play a central role in coping with low phosphorus stress and improving phosphorus use efficiency in wheat, and in-depth studies of their functions provide an important theoretical basis for solving the problems of low phosphorus stress in agricultural production. By regulating the expression and function of phosphate transporters, we can not only improve the yield and quality of wheat in low-phosphorus soils, but also reduce the amount of phosphorus fertilizer applied, reduce the negative impact on the environment, and achieve the sustainable development of agriculture. Therefore, it is vital to identify and functionally analyze genes related to low Pi stress tolerance in wheat, to enhance effective Pi use, and achieve high and stable wheat yields. In this study, key genes related to efficient Pi use were explored in cultivated wheat to provide genetic resources and technical support for cultivating new low Pi-tolerant wheat varieties.

## 2 Materials and methods

### 2.1 The dentification and phylogenetic analysis of *PHT* genes in the wheat genome

*Arabidopsis* and rice *PHT* family protein sequences were retrieved from TAIR and RiceData genome databases, respectively. Wheat whole genome data, protein sequences, and annotation files were downloaded from the Ensemble Plant database. BLAST comparisons were conducted using TBTools software to retrieve homologous *PHT* genes from the whole wheat genome based on *Arabidopsis* PHT protein sequences and identify wheat *PHT* family members. HMMER files for the conserved structural domain in *PHT* genes were downloaded from the PFAM database. Query sequences, *PHT1* (PF00083), *PHT2* (PF01384), *PHT3* (PF00153), *PHT4* (PF07690), *PHT5* (PF07690 and PF03105) and *PHO1* (PF03124 and PF03105), were downloaded from PFAM and used to search for *PHT* family members in the Wheat Protein Data Bank (E value ≤ 1×^10-10^). To guarantee data precision, the sequences underwent further verification in NCBI-CDD (National Center for Biotechnology Information-Conserved Domains Database) and Inter Pro databases, which confirmed defining structural domain characteristics in each subfamily. Following this, sequences were classified using established criteria. Based on this approach, genes with identical sequences and those lacking *PHT* structural domains were excluded. *Arabidopsis* and rice PHT protein sequences were obtained from the NCBI (National Center for Biotechnology Information) database and aligned with wheat sequences using multiple sequence alignments (MEGA 10 software). Alignments were then used to construct a phylogenetic tree using the Neighbor-Joining (NJ) method with 1000 bootstrap repeats to represent evolutionary relationships in the *PHT* family. The resulting “Newick” tree file was then exported and displayed on the Chiplot website (**Supplementary Table S1** for URLs).

### 2.2 *TaPHT* physicochemical properties, subcellular localization, chromosomal distribution, and conserved motif and promoter sequence analyses

*TaPHT* physical and chemical properties were predicted using ProtParam, while hydrophilicity was assessed using ProtScale. Phosphorylation sites were identified using Server, while signal peptide and transmembrane regions were predicted using SignalP-5.0 and TMHMM, respectively. Additionally, secondary and tertiary protein structures and structural domains were predicted using SOPMA, Swiss-Model, and SMART.

SMART was used to sequentially predict secondary and tertiary protein structures and also structural domains, while subcellular localization was determined using WoLF PSORT. Gene density profiles were calculated using TBtools software, and chromosomal gene positions were visualized using wheat GFF3 files. *TaPHT* conserved motifs were analyzed using the MEME website. Genome sequences and GFF3 annotation files for *Arabidopsis* and barley (*Hordeum vulgare*. L) were downloaded from the Ensembl Plant website, and covariance between *TaPHT* genes was analyzed using One Step MCScanX. Co-linearity within *TaPHT* gene species was analyzed and beautified using the Advanced Circos function. A 2000 bp sequence upstream of the *TaPHT* start codon was identified as a promoter (TBtools) and submitted to the Plant-CARE website to predict cis-acting elements (type and number) in the promoter sequence (**Supplementary Table S1** for all URLs).

### 2.3 Plant materials and growth conditions

The low phosphorus tolerant spring wheat material SW2 and phosphorus sensitive spring wheat material SW14 were provided by the State Key Laboratory of Aridland Crop Science/Gansu Key Lab of Crop Improvement and Germplasm Enhancement, and were derived from the results of previous research in this laboratory (Li et al., 2023). Seed surfaces were disinfected in 70%–75% alcohol for 30-60 s, quickly removed, and seeds rinsed 3–5 times in distilled water for 30 s each time. Then, seeds were transferred to Petri dishes for 1 h, after which 20 seeds were sown in each dish with the ventral groove facing downward. After 4 d, seedlings showing good growth and uniform development were selected and transplanted into a 900 mL hydroponic incubator, further incubated for 2 d, and then transferred to an artificial climate chamber with 60% relative humidity. White fluorescent lamps provided light conditions (irradiance = 300 µmol/m²/s) and Pi treatments were conducted over a day-night cycle: 16 h of light (25°C) and 8 h of darkness (18°C). The improved Hoagland nutrient solution was used in the wheat hydroponics test and was replaced every 3 days. The nutrient solution contains a large number of elements: 4 mM Ca(NO_3_)_2_·4H_2_O, 5 mM KNO_3_, 1 mM NH_4_NO_3_, 2 mM MgSO_4_·7H_2_O; and trace elements: 200 μM Na_2_-EDTA, 200 μM FeSO_4_·7H_2_O, 10 μM H_3_BO_3_, 13μM MnSO_4_·H_2_O, 0.01 μM CuSO_4_·5H_2_O, 3 μM ZnSO_4_·7H_2_O, 0.02 μM (NH_4_)_6_Mo_7_O_24_·4H_2_O. Normal phosphorus (NP) treatment: 500 mM KH_2_PO_4_; and low phosphorus (LP) treatments of 6.25 mM KH_2_PO_4_ and 247 mM K_2_SO_4_. Samples were collected at different time points after transplantation (0, 0.25, 0.5, 1, 3, 7, 14, and 21 d) and excess water of wheat roots were removed by gently pressing with filter paper. Then, roots and shoots samples were separately placed into 5 ml freezing tubes and quickly frozen in liquid nitrogen. Some samples were used for RNA extraction and the remainder were stored at -80°C.

### 2.4 *TaPHT* qRT-PCR analysis under low and normal Pi treatments

To explore the positive correlation between *TaPHT* expression and phosphorus uptake under low and normal phosphorus conditions, total P concentration in plant samples was measured with 3 biological replicates for each treatment. The fresh roots and shoots were dried in an oven at 105 °C. In order to facilitate the determination of total P concentration by molybdate blue colorimetry, the dried samples were crushed according to the method designed by Li (Li et al., 2023) and digested with concentrated H_2_SO_4_ and H_2_O_2_.To investigate *TaPHT* genes expression in response to phosphate starvation, relative gene expression levels in SW2 and SW14 cultivars were compared using qRT-PCR under normal Pi (NP) and low Pi (LP) conditions. Total RNA was extracted using a Total RNA Extraction Kit for Polysaccharide-Polyphenol Plants (TIANGEN, Beijing, DP441). RNA quantity and concentration levels were measured using the UVITEC Multi-color Fluorescence/Chemiluminescence Gel Imaging Analysis System (UVITEC, UK) and the Pultton P100/P100+ Ultra-Micro Spectrophotometer. To synthesize cDNA, a qPCR RT Master Mix with gDNA Remover (TOYOBO, Japan) was used following manufacturer’s instructions. All cDNA primers are shown (**Supplementary Table S2**), with *TaActin* an internal reference gene. SYBR-Green Prime Script RT-PCR kits (Takara Biotechnology, Japan) were used on the QuantStudio™ 5 System Real-Time System for qRT-PCR analysis. The reaction system is shown (**Supplementary Table S3**). Comparative Cycle (Ct) threshold (ΔΔCt) analysis was performed in 0.2 mL 96-well plates. Thermal cycling parameters were as follows; Hold stage with initial denaturation at 95°C for 30 s, then PCR Stage with denaturation at 95°C for 5 s, annealing at 60°C for 30 s, and cycling 40 times. Melt Curve Stage with denaturation at 95°C for 15 s, annealing at 60°C for 60 s, and separation at 95°C for 15 s. Three biological replicates were performed per treatment, and gene expression levels were calculated as average signal intensity values from replicates using the Ct method. Data were represented as the mean ± standard deviation from replicates, with error bars representing the standard error. Differences in *TaPHT* expression after low-Pi treatment were assessed using least significant difference (LSD) tests (*P* < 0.05) using the GenStat 12^th^ Edition.

### 2.5 Protoplast transformation and subcellular localization

Based on the *TaPHT1;36* gene sequence in the wheat reference genome, specific primers were designed to amplify the full-length coding region (CDS) sequence using the cDNA of the phosphorus-efficient SW2 root system of wheat as a template. The vector pC1300-GFP was selected to construct the 35S::TaPHT1;36-GFP fusion protein expression plasmid, and the PEG-mediated method was used to transform rice protoplasts, and the subcellular localization signals were observed and photographed using a laser confocal microscope (Leica STELLARIS 8).

## 3 Results

### 3.1 *TaPHT* identification, phylogenetic classification, and physicochemical properties

**Figure 1:**
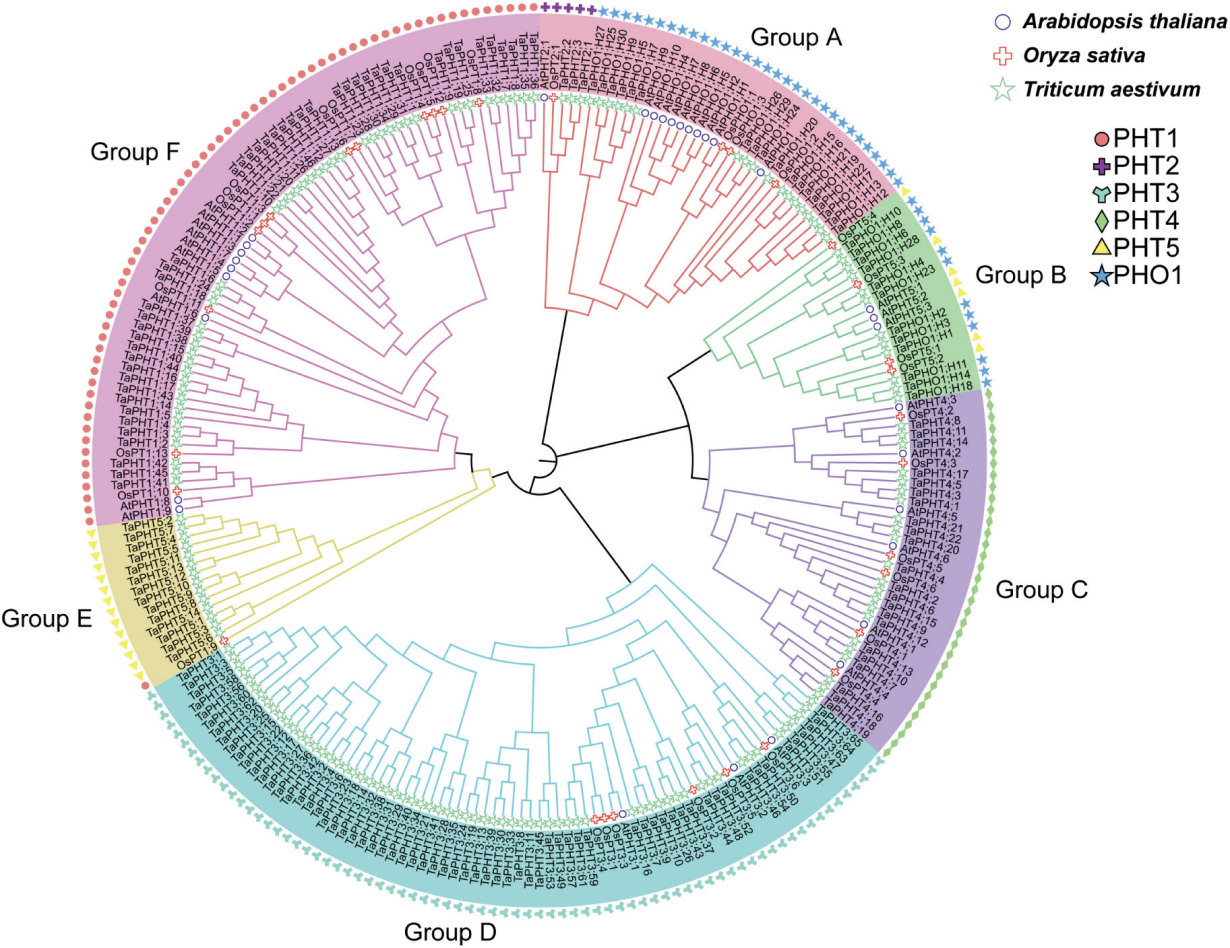
Phylogenetic analysis of PHT proteins in *Arabidopsis thaliana*, *Oryza sativa*, and *Triticum aestivum*.

To investigate the evolutionary relationships among *TaPHT* gene families, we performed multiple sequence alignments using protein sequences from 32 *Arabidopsis*, 33 rice, and 180 wheat. Subsequently, a phylogenetic tree was constructed using the NJ method. As shown (**Figure 1**), *Arabidopsis*, rice, and wheat *PHT* gene families were grouped into 6 branches, with 46 *TaPHT1* genes clustered in group F and 3 *TaPHT2*, 14 *TaPHT5*, and 30 *TaPHO1* genes clustered in groups A and B. *TaPHT2* was in a separate branch, and *TaPHT5* and *TaPHO1* were clustered in groups A and B due to the common PFAM number (PF03105); 22 *TaPHT4* genes were clustered in group C; and 65 *TaPHT3* genes were clustered in group D. *TaPHT* family genes were named according to *Arabidopsis* and rice, which were categorized for each subfamily and their location on the wheat chromosome (**Supplementary Table S5**).

Physicochemical TaPHT protein properties were analyzed using ProtParam (**Supplementary Table S4**). The following physiochemical ranges were identified; the encoded amino acid (aa) range was 276 (TaPHT3;37) to 855 (TaPHO1;H29); the molecular weight range was 29.9 (TaPHT3;37) to 97.1 (TaPHO1;H29) kDa; the theoretical pI range was 5.41 (TaPHT4;21) to 10.45 (TaPHT4;14); the instability index range was 21.52 (TaPHT3;4) to 52.97 (TaPHT4;22), the aliphatic index range was 76.75 (TaPHT3;40) to 118.65 (TaPHT5;5); and the GRAVY (Grand average of hydropathicity) range was -0.253 (TaPHT3;38) to 0.729 (TaPHT5;14). Subcellular localization predictions showed that 99/180 of TaPHT were localized to cell membranes.

### 3.2 *TaPHT* structure, chromosomal distribution, and conserved motif and promoter sequence analyses

**Figure 2:**
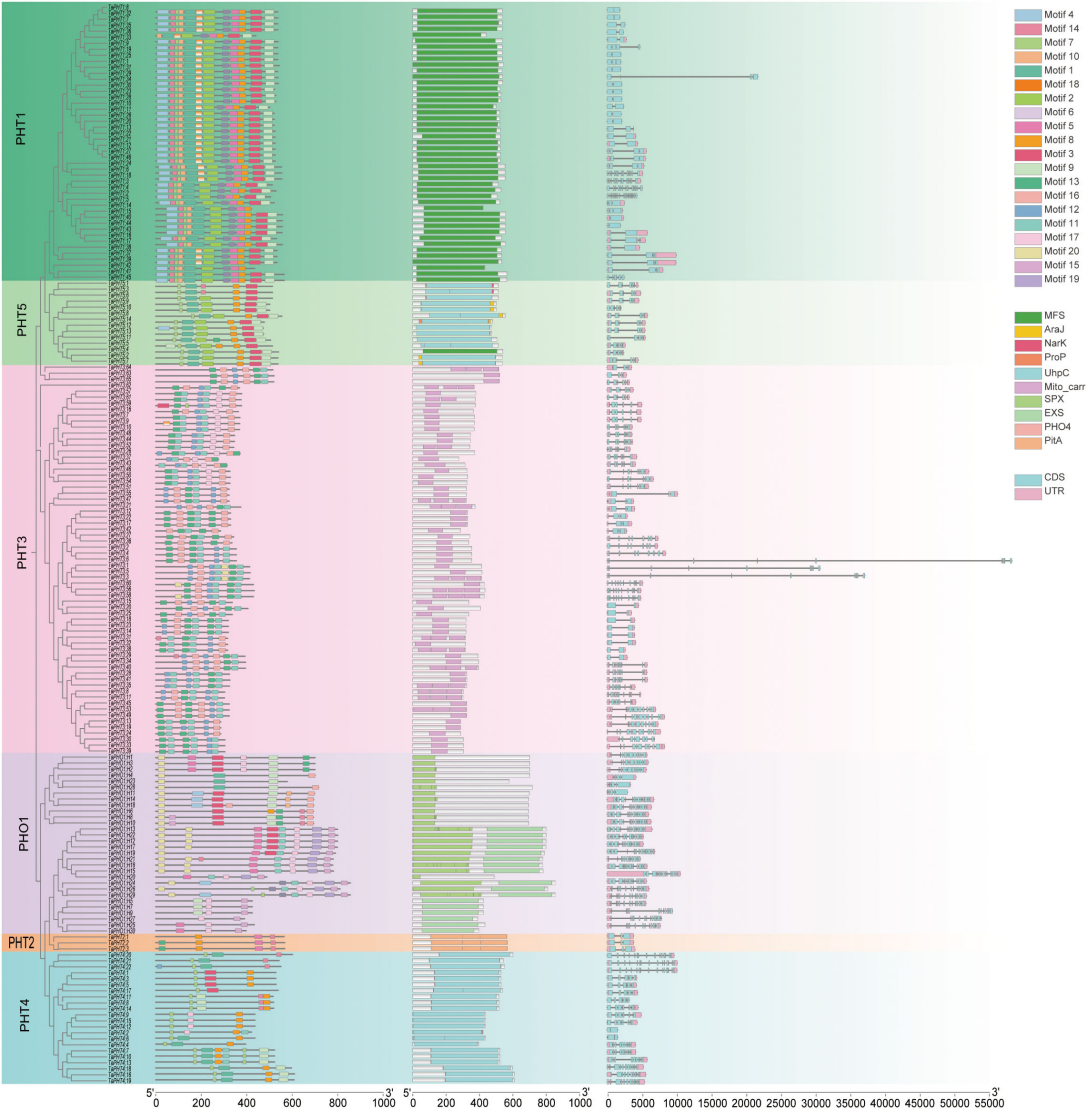
*TaPHT* structure analyses encompassing a phylogenetic analysis for each subfamily, the identification of conserved domains, and exon and intron distribution/counts for each *TaPHT* family member. Analyses also included an examination of conserved motifs in *TaPHT*. The scale (bottom of the graph) represents protein and nucleotide sequence lengths for each *TaPHT* member.

In genes, exon–intron structures are important evolutionary features that provide clues to gene family functions. To investigate *TaPHT* structural diversity in wheat, we evaluated conserved exon–intron organization. Using TBtools, 180 *TaPHT* coding sequences (CDS) were compared with corresponding genomic sequences to generate a *TaPHT* structure visualization map (**Figure 2**). All *TaPHT* genes had introns and exons. Genes with similar exon–intron structures and closer phylogenetic relationships showed more similar conserved structural domain organization. Exon and intron numbers were clearly partitioned according to subfamilies, with data showing different exon and intron numbers according to subfamily. The *TaPHT1* subfamily had 1-5 exons and 0-4 introns; the *TaPHT2* subfamily had 3 exons and 2 introns; the *TaPHT3* subfamily had 1–11 exons and 0-5 introns; the *TaPHT4* subfamily had 1-11 exons and 0-5 introns; and the *TaPHT5* subfamily had 1-11 exons and 0–10 introns. These findings suggested a high degree of variation among *TaPHT* genes, with comparable intron/exon distribution patterns within the same subfamily.

To structurally characterize *TaPHT*-conserved motif distribution, 20 conserved motifs were identified using protein sequence comparisons (MEME software) and labeled as 1-20, with MEME-8 having the highest E-value (**Table 1**; **Supplementary File S1**). Most *TaPHT1* subfamily members exhibited motifs 1-0, 14, and 18; the *TaPHT2* subfamily exhibited motifs 5, 10, 11, and 14; the *TaPHT3* subfamily included motifs 3, 11, 12, 16, 17, and 20; the *TaPHT4* subfamily included motifs 3, 7, 8, 9, 11, 14, and 17; the *TaPHT5* subfamily included motifs 1, 2, 3, 7, 8, 9, 13, and 14; and the *TaPHO1* subfamily included motifs 4, 9, 14, 15, 16, 17, 19, and 20.

**Table 1:** TaPHT protein family motif analysis.

Motif ID	Motif Sequence	Motif width	Sites	E-value
MEME-1	SVMATLCFFRFWLGFGIGGDYPLSATIMSEYANKKTRGAFIAAVFAMQGF	50	46	2.8e-2086
MEME-2	GPZADYVWRIILMLGAVPALLTYYWRMKMPETARYTALIAKNAKQAASDM	50	46	6.2e-1851
MEME-3	TFFFANFGPNSTTFIVPAEIFPARLRSTCHGISAAAGKAGAIIGAFGFLY	50	44	4.8e-1781
MEME-4	QLQVLSALDVAKTQLYHFTAIVIAGMGFFTDAYDLFCISLVTKLLGRIYY	50	45	7.2e-1800
MEME-5	WIPPAKTMSALEEVYRIARAQALIALCGTVPGYWF	35	44	1.4e-992
MEME-6	RHGLHLLGTTTTWFLLDVAFYSQNLFQKD	29	46	2.1e-970
MEME-7	GQLFFGWLGDKLGRK	15	94	5.8e-531
MEME-8	TVAFIDRIGRFWIQLMGFTMMTVFMLGLA	29	56	8.2e-878
MEME-9	PGIGVRNSLFVLAGCNLLGFLFTFLVPESKGKSLEELSGEN	41	42	1.9e-1016
MEME-10	SVYGMTLILMVLCSIASGLSF	21	46	5.1e-575
MEME-11	PGFYKGLSDAFPKIVRAEGFAGLYKGJGP	29	72	8.2e-683
MEME-12	SAGVIADTALCPLDLVKTRLQ	21	65	5.7e-488
MEME-13	CLLAGAAAGALAHTATTPLDVVKVRMQVQ	29	63	1.3e-549
MEME-14	PGSLPPNVAAAVNGVALCGTL	21	42	3.8e-466
MEME-15	LEIIRRGQWNFYRLENEHLNNVGKFRAVK	29	18	5.2e-357
MEME-16	GGFYRGLPLNILRVGPLTAIQFFTYETAK	29	62	6.0e-527
MEME-17	PKSZCSKHLQLGVSFLPYYWRGVQC	25	31	1.8e-352
MEME-18	GILAGGIVTJIVSSAFRHAFPAPAYQIDA	29	32	3.1e-463
MEME-19	QLYWDFVKDWGFFTPKSKNLWLRDDLILKNKFTYYISMMLN	41	12	5.0e-379
MEME-20	DQVPEWKGYYVBYKLLKKLIKQYKQQTQD	29	23	2.5e-351

### 3.3 Cis-acting elements and chromosome distribution

**Figure 3:**
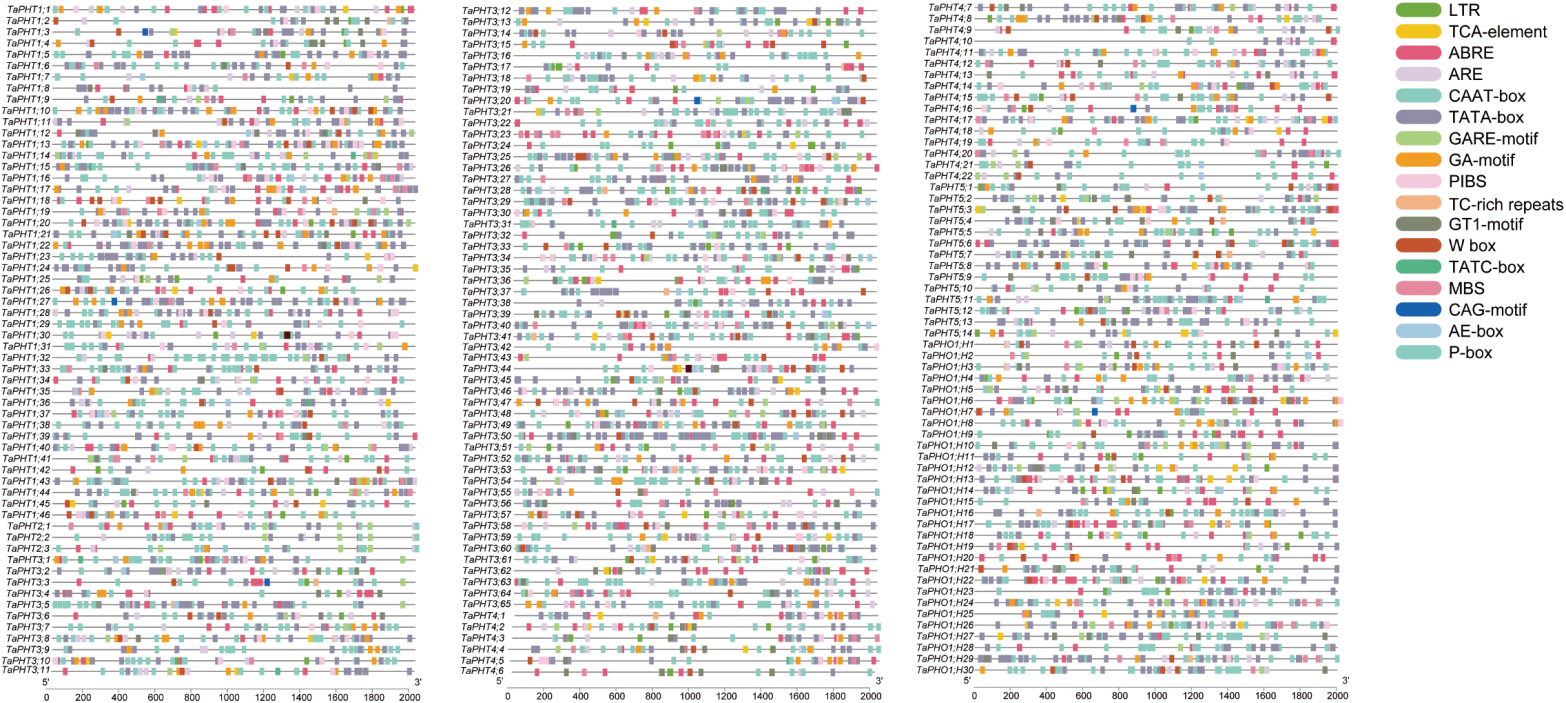
Cis-acting elements in the *TaPHT* family.

Cis-acting elements are crucial for gene transcription and expression as they regulate plant adaptation to different environments via different functions (Marand et al., 2023). To further identify *TaPHT* characteristics, 17 species and 7709 cis-acting elements in the *PHT* family 2000 bp promoter region were identified using PlantCARE, and element distribution related to growth and development, hormone regulation, and low-Pi regulation were analyzed. *TaPHT* promoter analyses showed that the majority of genes possessed multiple cis-acting elements which were related to stress, hormone, and light responses (**Supplementary Table S5**; **Figure 3**). Most family genes contained PIBS, W-box, and MBS elements, suggesting that these genes were transcriptionally induced by PHR1, WRKY, and MYB transcription factors under low Pi conditions to generate more Pi. These data suggested that *TaPHT* expression potentially regulated wheat growth and development, cytological processes, and adverse stress responses.

**Figure 4:**
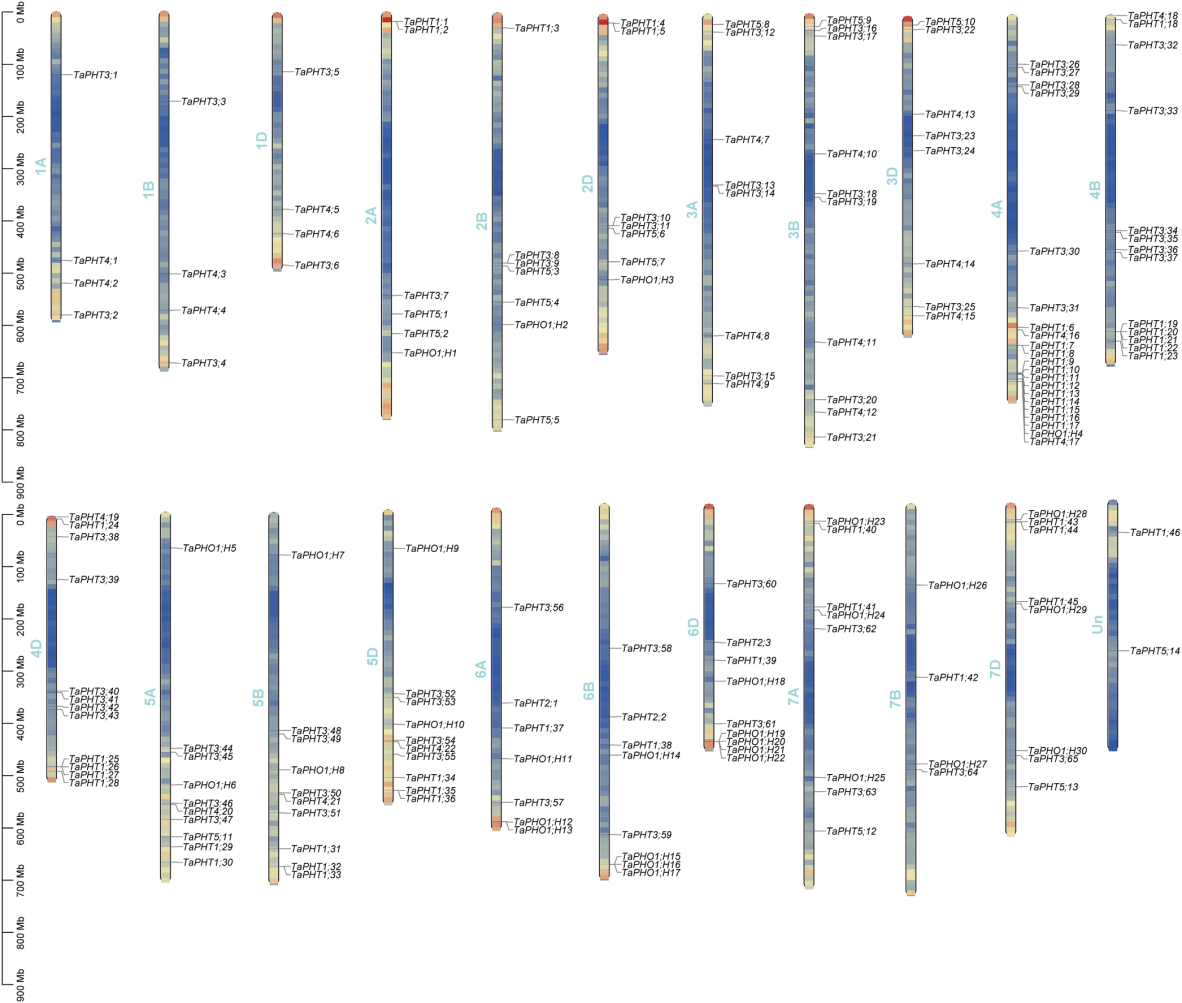
The chromosome distribution of *TaPHT* family members.

Based on the physical locations in the GFF3 file, the localization results showed that the 180 *TaPHT* genes on wheat chromosomes were distributed on 21 chromosomes of wheat (**Figure 4**). In the 3 homologous chromosome groups A, B and D of wheat, *TaPHT* genes were distributed on 21 chromosomes of wheat. The 180 *TaPHT* genes were not evenly distributed on the wheat chromosome, but were more densely distributed on the lower end of the chromosome. Except for 2 genes that were not located on chromosomes, the most genes were located on chromosomes 4A and 21; the number of genes located on chromosomes 1A, 1B, 1D and 7D is minimal. Among them, four genes were located on chromosomes 1A, 1B, 1D and 7D.

### 3.4 Intra- and inter-species collinearity analysis of *TaPHT* genes

**Figure 5:**
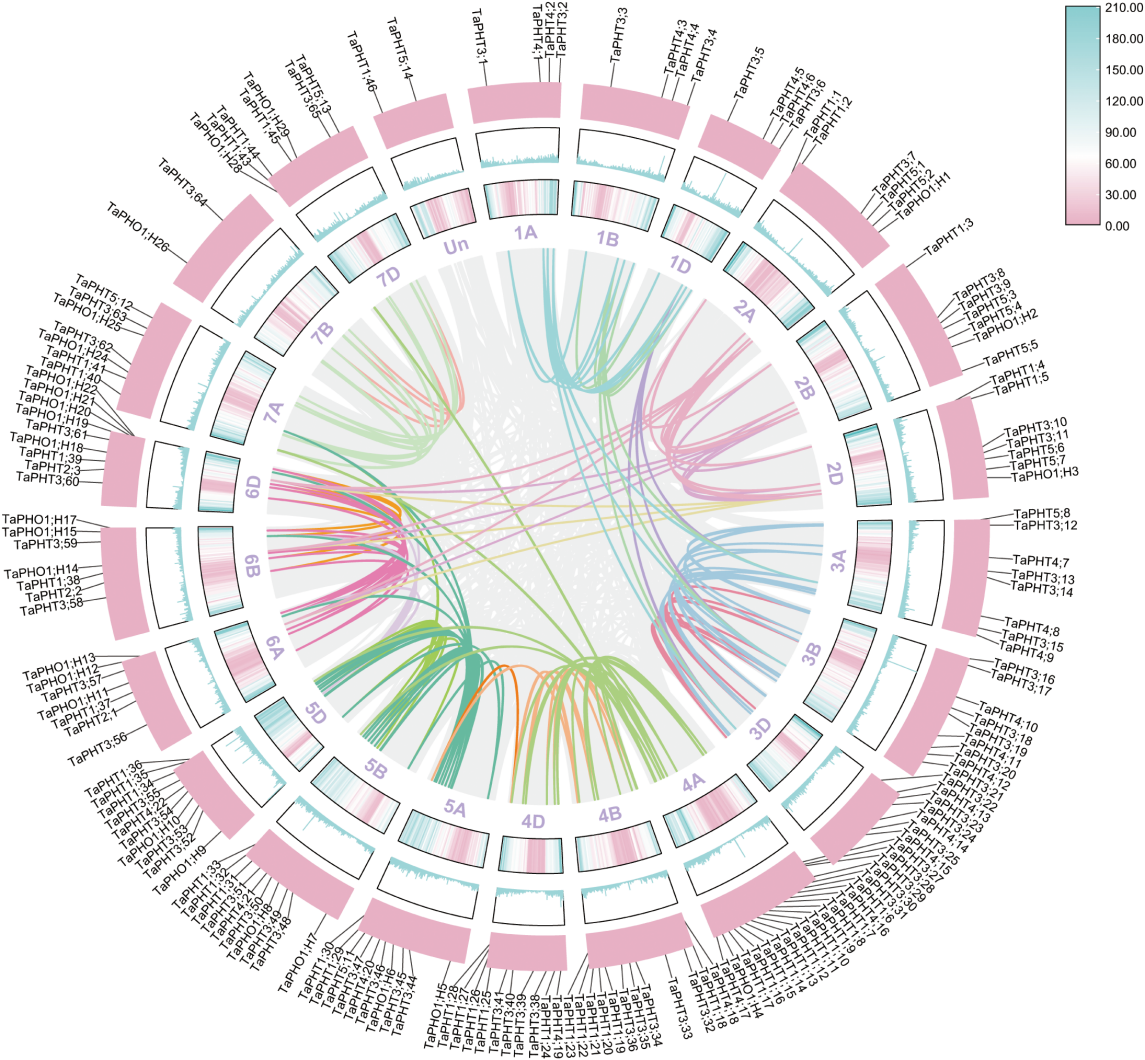
Collinearity from a species analysis of *TaPHT* genes.

To investigate evolutionary relationships among wheat, *Arabidopsis*, and barley *PHT* genes, intra- and inter-species collinearity analyses were performed using their genomes. We observed that the 180 *TaPHT* genes formed 159 collinearity gene pairs, with a denser distribution on chromosomes 4A–6D (**Figure 5**; **Supplementary Table S6**).

**Figure 6:**
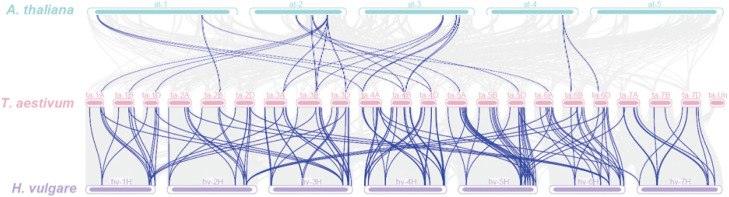
Interspecies *TaPHT* gene collinearity analysis with *Arabidopsis* and barley.

Wheat shared 25 covariates with *Arabidopsis*, mainly on *Arabidopsis* chromosomes 1–4 and wheat chromosomes 1A, 1B, 1D, 2B, 3A, 3B, 3D, 4A, 4B, 4D, 5A, 5D, 6A, 6B, and 6D, while it shared 142 covariates with barley, which were distributed on wheat chromosomes 1A–7D and barley chromosomes 1H–7H (**Figure 6**). These observations indicated that *TaPHT* genes exhibited obvious covariance with *Arabidopsis* and barley, with significant correlations. Thus, we hypothesized that these genes may have evolved from a same ancestor genome.

### 3.5 *TaPHT* expression patterns in different tissues and under low Pi stress

**Figure 7:**
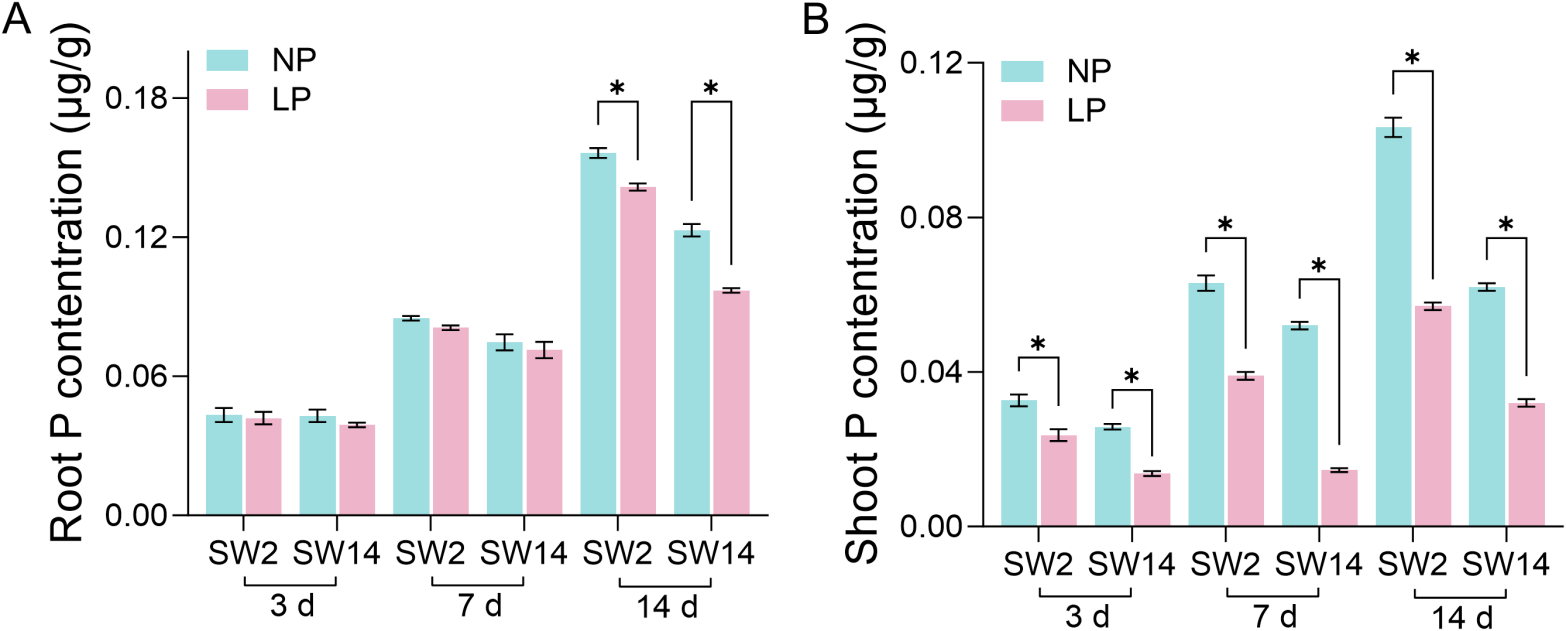
P concentration in root **(A)** and shoot **(B)** (μg/g). Student’s method was used to compare significant differences between LP and NP. *(*P* < 0.05) represents the significance of the difference between two treatments of the same variety at the same time point.

**Figure 8:**
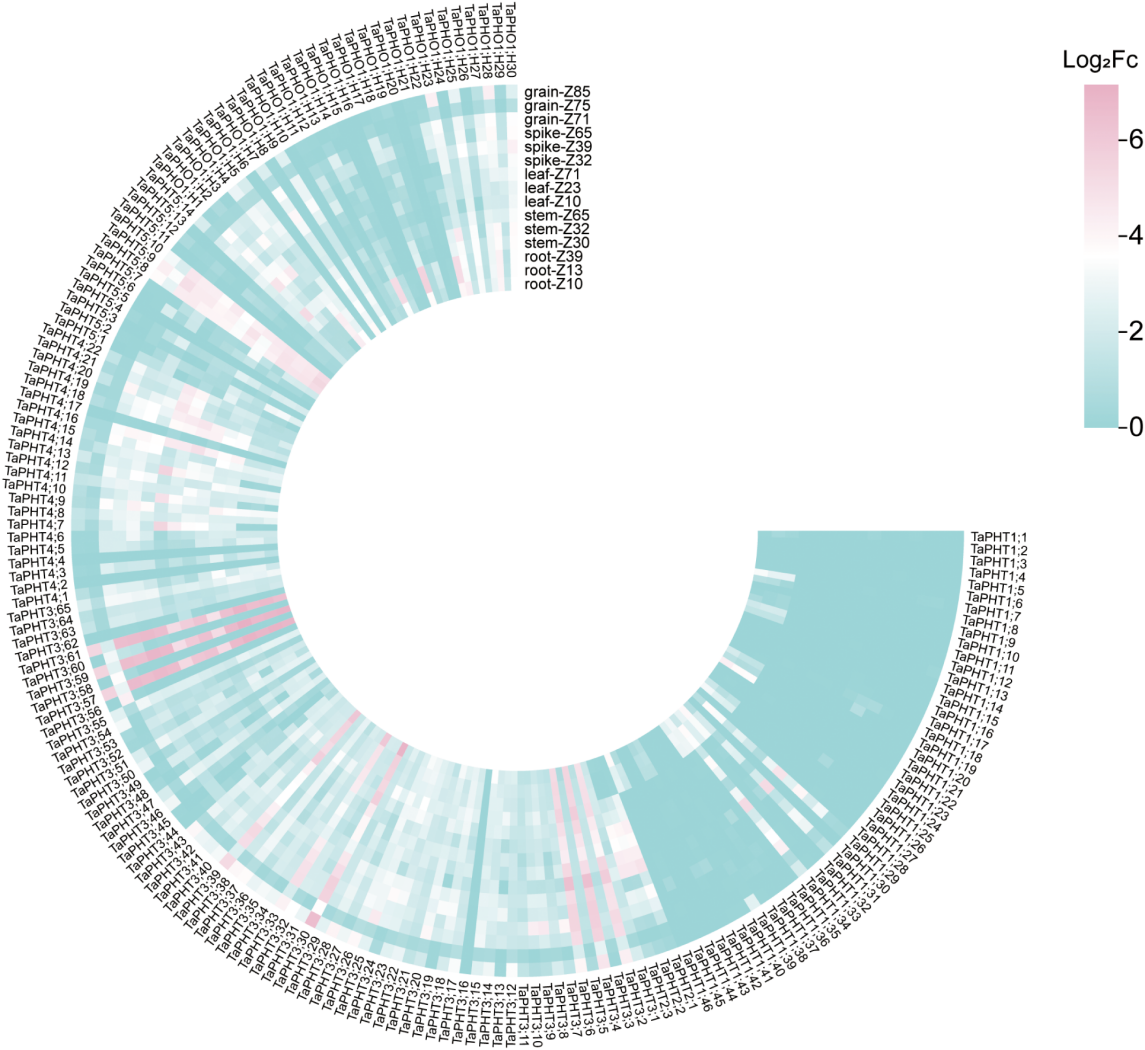
*TaPHT* expression patterns in different tissues. Grain_z71: seed 2d after anthesis; Grain_ z75: seed 14d after anthesis; Grain_z85: Seeds 20 days after flowering; Spike_z32: internode spike; Spike_z39: spike at visible flag leaf stage; Spike_z65: flowering spike; Leaf_z10: leaves of seedling stage; Leaf_z23: leaves in the three-tillering period; Leaf_z71: leaves 2d after flowering; Stem_z30: stem at heading stage; Stem_z32: stem interstitial; Stem_z65: flowering stem; Root_z10: root at seedling stage; Root_z13: root of trilobate stage; Root_z39: root of flag leaf visible period.

**Figure 9:**
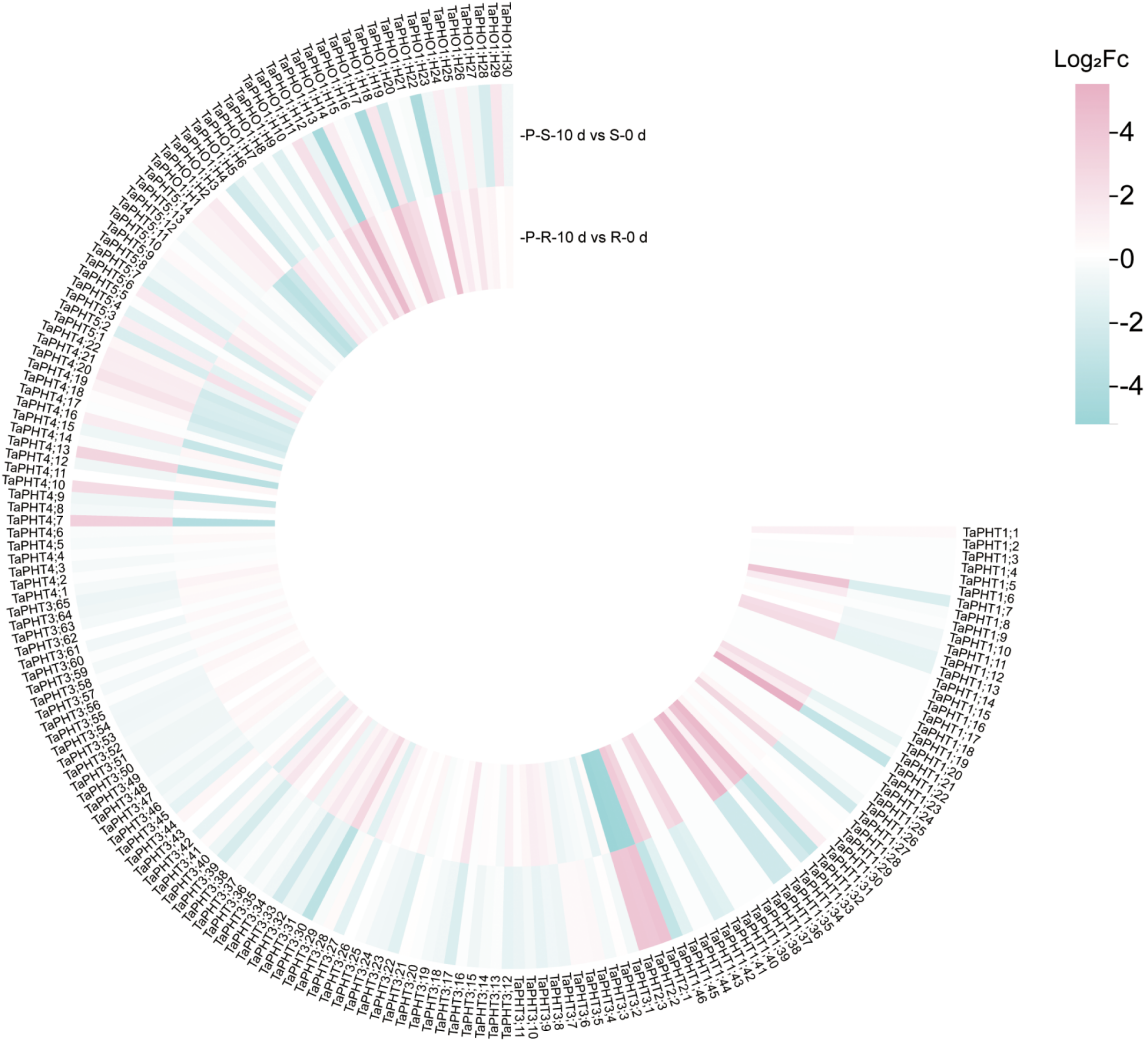
*TaPHT* expression patterns under low phosphorus (Pi) stress. -P-S-10 d: shoots with 10 d of low Pi stress; S-0 d: shoots with 0 d of low Pi stress; P-R-10 d: roots with 10 d of low Pi stress; R-0 d: roots with 0 d of low Pi stress.

In order to study the correlation between *TaPHTs* expression and phosphorus absorption under low and normal phosphorus conditions, we observed that SW2 and SW14 low phosphorus stress significantly decreased in wheat roots after 14 days of low phosphorus stress (**Figure 7**). In wheat shoots, there were significant differences on days 3-14, and the root P concentration of SW2 reached its peak on days 14 of low P stress (*P* < 0.05). The results showed that SW2 was more adaptable to low P stress than SW14 during 14 days of P stress. To understand *TaPHT* transcriptional level responses to low Pi stress, 180 *TaPHT* genes were searched against the Wheat Expression Database (Wheat Omics 1.0), combining tissue expression patterns in wheat roots, leaves, stems, spikes, and seeds, and gene expression under low Pi treatments (**Figures 8**, **9**). Among genes, *TaPHT3;2*, *TaPHT3;4*, *TaPHT3;6*, *TaPHT3;30*, *TaPHT3;33*, *TaPHT3;39*, *TaPHT3;57*, *TaPHT3;59*, *TaPHT3;61*, *TaPHT5;8*, *TaPHT5;9*, and *TaPHT5;10* were expressed in all tested tissues. Under low Pi stress, *TaPHT1* and *TaPHT3* subfamilies were up-regulated in roots and down-regulated in shoots, whereas *TaPHT2* and *TaPHT4* subfamilies were up-regulated in shoots and down-regulated in roots, while most of the *TaPHT5* subfamily was up-regulated in shoots, and most of the *TaPHO1* subfamily was up-regulated in roots. Among the six subfamilies, the highest root expression level was observed for the *TaPHT1* subfamily, while the highest expression level in shoots was observed for the *TaPHT2* subfamily.

We next selected 3–5 genes in each *TaPHT* subfamily based on predicted expression results, and examined the expression of 30 *TaPHT* genes in wheat roots and shoots, under low Pi stress, by qRT-PCR. Six genes were not expressed or abnormally expressed, with the remaining 24 analyzed in roots and shoots.

In the *TaPHT1* subfamily, 0-3 d was the initial response period. Wheat root *PHT1* family members rapidly responded to low Pi stress with increased relative expression, which kept rising as the stress continued, then slowly decreased from 14 - 21 d. *TaPHT1;36* in roots had the highest relative expression of 114.727 at 7 d and then stabilized, suggesting its role in Pi uptake under long - term low Pi stress. The LSD value (**Supplementary Figure S2**) indicated that *PHT1* family members in wheat roots responded rapidly to low Pi stress, with increased relative expression. The distance between the two points in **Figure 9** is greater than the distance of the LSD line segment, indicating that there is a significant difference based on LSD (*P* < 0.05). The specific LSD values are shown in **Supplementary Table S7**. *TaPHT2* subfamily levels in roots were slightly up-regulated in the 0–21 d period of low Pi stress, then gradually adapted to the low Pi environment after peaking at 7 d. In the *TaPHT3* subfamily, except for *TaPHT3;36* whose relative expression peaked at 2.207 at 7 d, the relative expression of all other genes increased slowly, suggesting that physiological stress caused by low Pi could be alleviated by slightly increased *TaPHT3* expression. Under low Pi conditions, wheat optimizes Pi uptake, transport, and partitioning by adjusting the expression of *TaPHT4* family members to adapt to the low Pi environment. Among the 5 *TaPHT4* subfamily genes, *TaPHT4;10* and *TaPHT4;13* were highly expressed in wheat roots, suggesting that up-regulated *TaPHT4* helped plants increase Pi uptake in low Pi environments. *TaPHT5* was slightly up - regulated in roots, with *TaPHT5;9* peaking at 3.841 after 7 d and others stabilizing. In the *TaPHO1* subfamily, *TaPHO1;H11* had higher relative expression in SW14 than SW2, likely due to cultivar differences. With low Pi stress, *TaPHO1* expression in roots gradually stabilized and reduced Pi - nutrition dependence. Except for *TaPHO1;H11*, the SW2 cultivar had higher relative expression in the *PHT* gene family in wheat roots than SW14. These results suggested that up-regulated *TaPHT* genes were possibly involved in phosphate uptake when soil phosphate levels were limited, thus alleviating physiological stresses caused by low Pi.

In wheat shoots, relative *TaPHT1;36* expression was close to 3, while relative *TaPHT1* subfamily expression, except *TaPHT1;36*, was in the 0.2-1.2 range over the 0-21 d period (**Supplementary Figure S3**). This, together with relative expression in wheat roots, indicated that *TaPHT1* subfamily genes were expressed in wheat roots. *TaPHT2* subfamily expression in early low-Pi treatment in shoots had no significant change, but with longer treatment, it was up - regulated, with *TaPHT2;1* peaking at 64.985 at 7 d and then stabilizing to adapt to low Pi stress and improve Pi transport to leaves. For the *TaPHT3* subfamily, mainly involved in Pi transport and distribution, except for *TaPHT3;36*, other *TaPHT3* genes had low relative expression in shoots. *TaPHT4* subfamily expression didn’t change much at first in shoots, with *TaPHT4;20* peaking at 7 d and then decreasing. *TaPHT5* subfamily expression decreased, and *TaPHT5;7* was higher in SW14 than SW2 after 3 d, likely due to factors like Pi transport and leaf physiology. In the *TaPHO1* subfamily, *TaPHO1;H11* had much higher relative expression. Shoots quickly sensed low Pi and *TaPHO1* subfamily expression increased with treatment time, peaking at 7 d, then declined at late stages as wheat adapted and possibly initiated other metabolic pathways. Wheat shoots are not the main direct exposure sites to low P stress, so their gene expression responses are relatively delayed, but overall up-regulated *TaPHT* family expression in wheat shoots was higher in the Pi-efficient material SW2.

In conclusion, qRT-PCR analysis of 24 low phosphorus responsive *TaPHT* genes showed that in the low phosphorus tolerant material SW2, the relative expression of the *TaPHT1;36* gene in wheat roots reached a peak of 114.727 at 7 d and then reached a more stable state with high and more stable relative expression. Thus, this further confirmed the subcellular location of *TaPHT1;36*.

### 3.6 Characterizing the subcellular localization of the low Pi tolerance candidate *TaPHT1;36*

**Figure 10:**
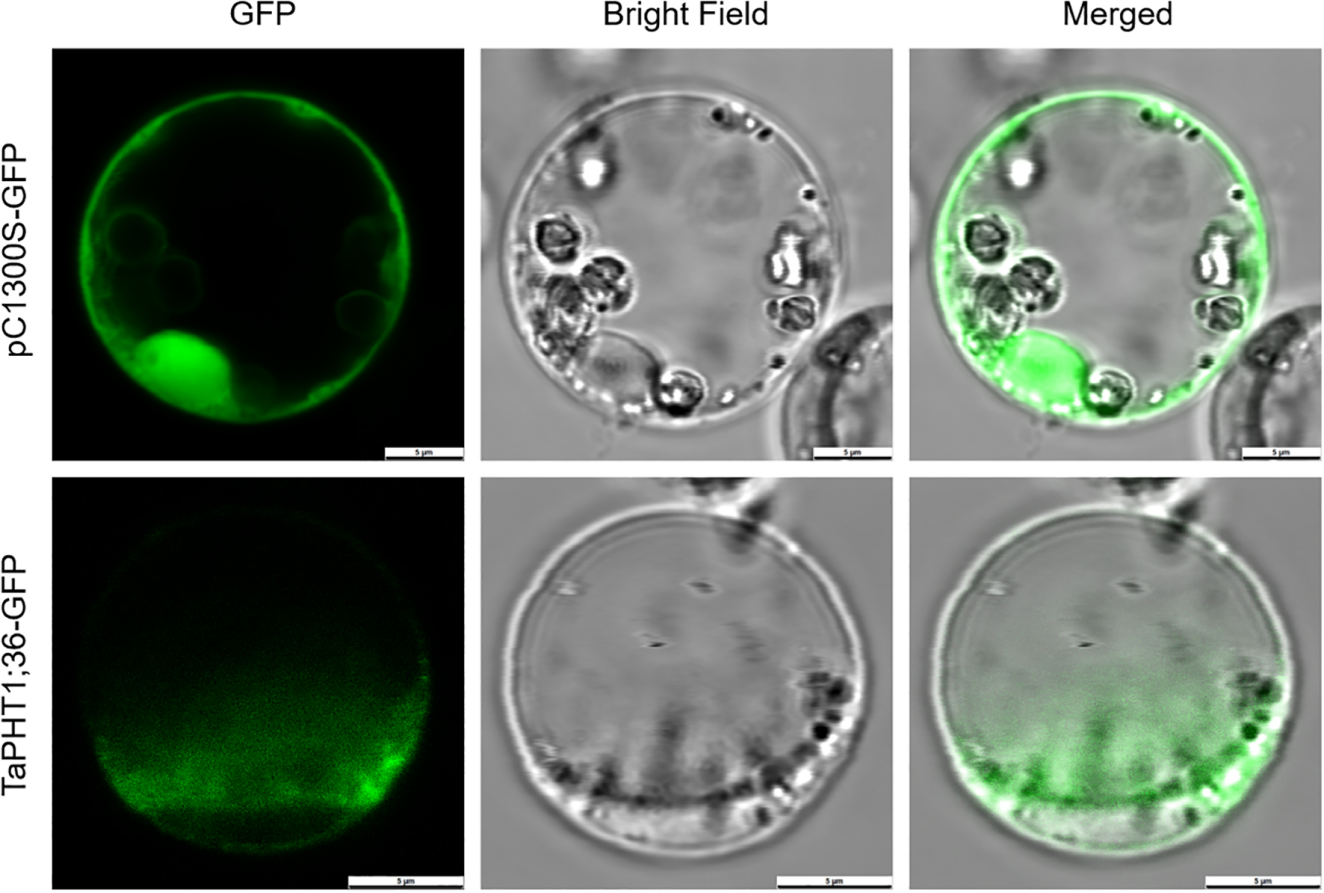
TaPHT1;36 subcellular localization after transient expression in rice protoplasts. Scale 5 μm.

To validate the results of online prediction of subcellular localization of gene expression proteins and to investigate the subcellular localization of TaPHT1;36 protein, the TaPHT1;36-GFP fusion protein expression vector was constructed and the empty vector pC1300S-GFP was used as a control. According to the results of rice protoplasts, GFP green fluorescence signals were detected throughout the cells of GFP empty control protoplasts with no obvious organelle localization specificity, and the GFP green fluorescence in the ligated target protein TaPHT1;36 was mainly distributed in the plasma membrane (**Figure 10**). The results indicate that TaPHT1;36 protein is localized to the plasma membrane, which is consistent with previous studies and online predictions.

## 4 Discussion

Phosphate ore is a non-renewable resource, and with the growing demand for phosphate fertilizers in global agriculture, phosphate resources are increasingly at risk of depletion (Chowdhury et al., 2017). Pi is a vital macronutrient required for plant growth and development, and is absorbed by roots via PHT proteins, which have key roles supporting overall plant growth and development (Zhang et al., 2016). Reduced Pi availability, increased Pi fertilizer costs, and decreased plant Pi efficiency have highlighted a need to investigate wheat PHT protein families. Cereals are important Pi reservoirs for plant growth and development (Godfray et al., 2010). Wheat is a globally important food crop, occupies a prominent position in global cereal production, and uses a large amount of Pi, which is stored in seeds as PA (Phosphatidic acid) (Wang et al., 2019a). Increasing the expression efficiency of *TaPHT* under low phosphorus stress is a key factor in promoting their yield and quality. Currently, the sequencing of the full-length wheat genome is providing important genetic resources for further functional genomics research and genetic improvements in crops. Considering the close evolutionary relationship between wheat and its homologs in *Arabidopsis* and rice, we hypothesized that *TaPHT* genes could exhibit similar functions. To do this, we successfully identified *TaPHT* family members and comprehensively analyzed their phylogenetic relationships and expression patterns across various tissues and growth stages in wheat and in different cultivars during various growth stages under low Pi stress. This study not only lays the foundation for investigating the molecular mechanisms of inorganic phosphate transport in response to abiotic stress, but also has important implications for improving wheat yield under low phosphorus stress and addressing global challenges to sustainable agriculture.

### 4.1 The identification of *TaPHT* families

In dicotyledonous plants, the number of *PHT* families varies, e.g., 23 *PHT* genes occur in sugarcane and 73 occur in *S. polyrhiza* (Mudge et al., 2002; Zhao et al., 2021b). Previous research involving the genome-wide identification and expression analysis of the *PHT1* subfamily in wheat identified 36 *TaPHT* genes. It was noted that heterozygous hexaploid wheat expressed significantly more *PHT1* genes when compared to diploid cereals like barley and rice, and not all *PHT1* genes in wheat were isolated (Teng et al., 2017). A previous genome-wide *PHT*-based study identified only 23 wheat *PHT* (mainly *TaPHT1*–*TaPHT4*) and focused on tissue-specific transcriptional profiling and associations with phosphate partitioning in cereals (Shukla et al., 2016). Thus, detailed studies of the wheat *PHT* family based on de novo genome sequencing have not been reported. However, recent advances in wheat genome sequencing have enabled extensive bioinformatics analyses of the *TaPHT* family. In our study, we extended these findings based on 180 *TaPHT* genes identified in the latest wheat genome, including 46 *PHT1*, three *PHT2*, 65 *PHT3*, 22 *PHT4*, 14 *PHT5*, and 30 *PHO1* genes. Proteins with high homology in a class/subfamily may have similar activities, so to characterize putative *PHT* sequences, we predicted conserved PHT protein structural domains using the NCBI -CDD (Liu et al., 2021). Consistent with previous studies, conserved structural domains were found in all proteins which were confirmed as typical PHT proteins (Aslam et al., 2022).

Notably, in contrast to the 46 *PHT1* genes identified in wheat, 9 *PHT1* genes were previously identified in *Arabidopsis*, 8 of which were expressed in roots and had key roles in Pi harvesting in low and high Pi environments. Also, 13 genes were identified in rice, where they had redundant roles in root-to-shoot Pi transport (Mudge et al., 2002; Ruili et al., 2020; Wan et al., 2020). Single genes for *PHT2* were identified in many plants, including tomato (Rui et al., 2023), potato (Liu et al., 2017), and *C. annuum* (Ahmad et al., 2021). In contrast, 3 *PHT* genes (*TaPHT2;1*, *TaPHT2;2*, and *TaPHT2;3)* located on different chromosomes (6A, 6B, and 6D) and encoding 564–567 aa with predicted molecular weights of 58.2-58.8 kDa were detected in wheat, similar to a previous report showing that *AtPHT2.1* cDNA encoded 587 aa with a predicted molecular weight of 61 kDa (Daram et al., 1999). So far, at least three *AtPHT3* genes have been identified in *Arabidopsis*, 6 *PHT3* genes in rice, and here, we identified 65 *PHT3* family genes in wheat, evenly distributed on 21 chromosomes, wheat 1A-6D (Wang et al., 2017, 2020). Additionally, 6 *PHT4* family genes were characterized in *Arabidopsis*, 6 in rice, and 22 in wheat (Ruili et al., 2020; Wang et al., 2017). It is noteworthy that *PHT5* family genes have not been identified in most plants, including potato (Liu et al., 2017), sugarcane (Murugan et al., 2022), and poplar (Zhang et al., 2016). Previous studies have reported three *PHT5* genes in *Arabidopsis* and four in rice, and in this study, we identified 14 in wheat (Wang et al., 2017). Additionally, 10 genes homologous to *AtPHO1* occur in the *Arabidopsis* genome and only three *PHO1* genes in rice, while 30 *PHO1* genes were identified in wheat in this study (Secco et al., 2010; Wang et al., 2004).

Cis-acting elements in promoter regions are important for the regulation of stress-related gene expression and to help plants resist abiotic and biotic stresses (Li et al., 2021). Cis-regulatory elements also have key roles in gene transcription and subsequent expression, while their diverse functional regulatory mechanisms significantly enhance plant adaptation to different environmental conditions (Li et al., 2019; Wang et al., 2021). Previous studies have detailed cis-elements in *PHO* family members in wheat; however, cis-elements in all *PHT* family members in wheat remain to be identified (Shukla et al., 2016). *TaPHT* genes are transcriptionally induced at low Pi levels, probably because they contain P1BS (PHR1 binding site), W-box (WRKY binding site), and MBS (MYB binding site) in their promoter regions, which are involved in *PHT* gene regulation. These transcription factors are highly distributed in the upstream 2000 bp regions of *TaPHT* genes (Nash et al., 1990; Teng et al., 2017; Zhao et al., 2021a). In *Arabidopsis*, functional *PHT* expression is initiated downstream by binding to cis-acting elements in P1BS (GNATATNC), where phosphate starvation-regulated response factors have important roles in Pi starvation signaling (Zhang et al., 2024). The P1BS cis-acting element is present in the barley *PHT1* promoter, with multiple copies in other genes (Schünmann et al., 2004). The TATA-box is a key component sequence in eukaryotic promoters that determines the onset of gene transcription, with increased TATA-box expression observed in *Arabidopsis* promoters in response to low Pi stress (Hammond et al., 2003). ABRE is a well-known cis-acting element that facilitates abscisic acid-dependent signaling, which has important effects on plant growth, development, and adversity responses (Baker et al., 1994) LTR is a DNA sequence in the plant genome involved in regulated low temperature responses (White et al., 1994). Additionally, TaPHT contain various responsive elements, including those related to light, antioxidants, gibberellin, drought stress, salicylic acid, and general stress responses. Therefore, these cis-acting elements are required to regulate plant gene expression and manage processes such as plant growth, development, and stress responses by reacting to different biotic and abiotic signals. Thus, cis-acting elements in *TaPHT* genes, along with their potential roles regulating gene expression, suggest that *TaPHT* genes have key roles in stress responses in wheat.

### 4.2 *TaPHT* family expression pattern analysis

Among the 180 *TaPHT* genes screened against the public transcriptome database, 24 showed significantly higher expression in roots or aerial parts under low Pi stress conditions, suggesting important roles in Pi transport and regulation. Pi transport protein genes are induced in plants in response to Pi depletion, processes also reported in *Arabidopsis*, rice, *B. napus*, poplar, maize, barley, and other plants (Mudge et al., 2002; Rae et al., 2003; Shukla et al., 2016; Teng et al., 2017; Wan et al., 2020; Wang et al., 2014).

*PHT* family genes have different expression patterns in different tissues. Our qRT-PCR analysis of *TaPHT* showed that *TaPHT1* was mainly expressed in roots, with previous studies showing that *TaPHT1.1/9*, *TaPHT1.2*, and *TaPHT1.10* were mainly expressed in roots, and that low-Pi treatments in hydroponic cultures significantly induced *TaPHT1* expression (Teng et al., 2017). Additionally, most *PtPHT* genes (from poplar) were highly expressed in *P. simonii* roots, whereas *PtPHT1.5* and *PtPHO9* were highly expressed only in *Populus tremula* roots and suckers (Zhang et al., 2016). *TaPHT2* was predominantly expressed in shoots, consistent with previous studies, but most reported *PHT2;1* genes were predominantly expressed in green tissues (Rausch et al., 2004; Versaw and Harrison, 2002), and all *AtPHT* genes showed detectable expression levels in roots. *MdPHT3;3* (apple) showed high expression in roots, stems, and young fruits. Similarly, in the *PHT3* subfamily of *Populus*, *PtPHT3.1a* is low expressed in root, freshly expanded and mature leaves, and high expressed in expanding young leaves. *PtPHT3.1b* is highly expressed in root and expanding young leaves, but low in freshly expanded and mature leaves. The expression of *PtPHT3.2a* was low in root, freshly expanded and mature leaves. There was no significant change in the expression of expanding young leaves. *PtPHT3.2b* is highly expressed in root and expanding young leaves. The expression was low in freshly expanded and mature leaves. *PtPHT3.3a* is highly expressed in root, freshly expanded and mature leaves. The expression was low in expanding young leaves. *PtPHT3.3b* is highly expressed in root, expanding young and mature leaves. There was no obvious change of expression in freshly expanded leaves (Zhang et al., 2016). In our study, *TaPHT3;36* was more highly expressed in wheat shoots and less in roots, which was possibly due to species differences (Sun et al., 2017). In the *TaPHT4* subfamily, relative *TaPHT4;10* and *TaPHT4;13* expression was high in wheat roots, while *TaPHT4;20* was high in wheat shoots. Previous studies reported that *AtPHT4;1* and *AtPHT4;4* had the highest expression levels in leaves (Guo et al., 2008), and *OsPT4;1*, *OsPT4;4*, and *OsPT4;5* were highly expressed in leaves, but *OsPT4;2* was highly expressed in roots and stems at nodal growth stages, suggesting that *PHT4* expression is varied across different species and tissues (Ruili et al., 2020). *TaPHT5* and *TaPHO1* were expressed in both shoots and roots, with levels up-regulated for a short period from 0–3 d, and then followed by relatively high expression levels at 7 d or 14 d in sustained Pi stress responses Similar to the low standing *Physcomitrella patens*, *PpPHO1;1* and *PpPHO1;7* expression was progressively increased over a 14 d Pi deficiency treatment period (Wang et al., 2007). In our study, relative *TaPHT* expression was up-regulated in both aboveground and roots by low Pi stress in SW2 and SW14 cultivars, consistent with observations in hairy camelina, maize, and three other grass crops (Xu et al., 2018). These results indicated that *TaPHT* family genes maintained normal plant growth and Pi homeostasis under low Pi stress.

The *PHT1* family is a major Pi transporter family in plants and facilitates Pi transport from the soil to the plant. Under low Pi stress, plants may respond to Pi deficiency by up-regulating *PHT1* family members to increase Pi uptake and transport capacity. Relative *TaPHT1;36* expression in wheat roots was high and stable among the *TaPHT* family in the low Pi tolerant SW2 cultivar (by qRT-PCR), further confirming its subcellular location. Importantly, subcellular location prediction databases often differ and thus require validation based on experimental data (Gillani and Pollastri, 2024). In this study, *Arabidopsis* was transiently transformed with TaPHT1;36-GFP and empty control pC1300S-GFP vectors, examined under laser confocal microscopy, which showed that fluorescence signals from the empty vector were localized to the nucleus, cell membrane, and cytoplasm, while TaPHT1;36-GFP signals were localized to the plasma membrane. These observations suggested TaPHT1;36 involvement in Pi transport, consistent with subcellular localization data from Gene Sequences Online, which is of great importance to verify its response to low Pi stress. The BnPHT1-GFP fusion protein was constructed in *B. napus*, a kale-type oilseed rape, with the 35S:BnPht1;4-GFP fusion construct introduced into onion endoepidermal cells using the PDS-1000 helium biobody device. Fluorescence signals were localized to the cell periphery using confocal laser microscopy, showing that the BnPHT1 protein was located to the plasma membrane, consistent with TaPHT1;36 (Ren et al., 2014).

## 5 Conclusion

We comprehensively analyzed the *TaPHT* family in terms of gene structure, conserved motifs, cis-acting elements, and expression patterns. We identified 180 *TaPHT* genes, which were classified into 6 groups (*TaPHT1*–*TaPHT5* and *TaPHO1*) based on phylogenetic relationships. Cis-acting element analysis of *TaPHT* promoters revealed multiple Pi-responsive elements. qRT-PCR analysis of *TaPHT* genes showed that relative expression in the low-Pi-tolerant wheat SW2 cultivar was higher than in the Pi-sensitive SW14 cultivar under low-Pi stress. We identified *TaPHT1;36* which had the highest relative expression levels in wheat roots, which will be further analyzed at physiological and molecular levels in future work. Our whole genome *TaPHT* analysis has provided a platform for screening and verifying genes during low Pi stress and uptake, which may improve Pi use efficiency for greener agricultural production.

## Data Availability

The original contributions presented in the study are included in the article/**Supplementary Material**. Further inquiries can be directed to the corresponding author.
